# Shining a light on elusive lynx: Density estimation of three Eurasian lynx populations in Ukraine and Belarus

**DOI:** 10.1002/ece3.10688

**Published:** 2023-11-09

**Authors:** Stefano Palmero, Adam F. Smith, Svitlana Kudrenko, Martin Gahbauer, Dominik Dachs, Kirsten Weingarth‐Dachs, Irina Kashpei, Dmitry Shamovich, Denys Vyshnevskiy, Oleksandr Borsuk, Kateryna Korepanova, Andriy‐Taras Bashta, Rostyslav Zhuravchak, Viktar Fenchuk, Marco Heurich

**Affiliations:** ^1^ Department of Wildlife Ecology and Management, Faculty of Environment and Natural Resources University of Freiburg Freiburg Germany; ^2^ Department of National Park Monitoring and Animal Management Bavarian Forest National Park Grafenau Germany; ^3^ The Frankfurt Zoological Society Frankfurt Germany; ^4^ Faculty of Technology, Natural Sciences and Maritime Sciences, Department of Natural Sciences and Environmental Health University of South‐Eastern Norway Bø Norway; ^5^ Meles Wildbiologie Großraming Austria; ^6^ Habitat – Wildlife Services Großraming Austria; ^7^ Sosnovy Bor Vitebsk Region Belarus; ^8^ Chornobyl Radiation and Ecological Biosphere Reserve Ivankiv Ukraine; ^9^ Institute of Ecology of the Carpathians National Academy of Sciences of Ukraine Lviv Ukraine; ^10^ Skolivski Beskydy National Park Skole Ukraine; ^11^ Faculty of Applied Ecology, Agricultural Sciences and Biotechnology Inland Norway University of Applied Sciences Evenstad Norway

**Keywords:** camera trapping, Carpathians, Chornobyl Exclusion Zone, density estimation, Eurasian lynx, Polesia, spatial capture–recapture

## Abstract

The Eurasian lynx is a large carnivore widely distributed across Eurasia. However, our understanding of population status is heterogeneous across their range, with some populations isolated that are at risk of reduced genetic variation and a complete lack of information about others. In many European countries, Eurasian lynx are monitored through demographic studies crucial for their conservation and management. Even so, there are only rough and fragmented population assessments from Ukraine and Belarus, despite strict protection in both countries and their importance for lynx connectivity across Europe. We monitored lynx from October 2020 to March 2021 and used camera trapping in combination with spatial capture–recapture (SCR) methods in a Bayesian framework to provide the first SCR density estimation of three lynx populations across Ukraine and Belarus, including the Ukrainian Chornobyl Exclusion Zone, southern Belarus and the Ukrainian Carpathians. Our density estimates varied within our study areas ranging from 0.45 to 1.54 individuals/100 km^2^. This work provides a substantial scientific component to the overall understanding of lynx conservation for a region where only broad information is available and opens the doors for further large‐scale monitoring and trend assessments. The crucial information we provide can greatly enhance the range‐wide assessments of the status of this protected species. We also discuss the implications for Eurasian lynx conservation, despite the geopolitical realities impacting species monitoring in the region. Our work serves as a baseline, not only for future conservation interventions but also to evaluate the effects of disturbance and threats to these protected populations.

## Graphical abstract

We estimated density of three lynx populations in previously unsurveyed areas in Ukraine and Belarus, including the Chornobyl Exclusion Zone, using camera trapping and spatial capture–recapture (SCR) methods. Our results can help the range‐wide assessments of the species status and serve as a baseline for future conservation interventions, as well as to evaluate the effects of disturbance and threats to these protected populations.

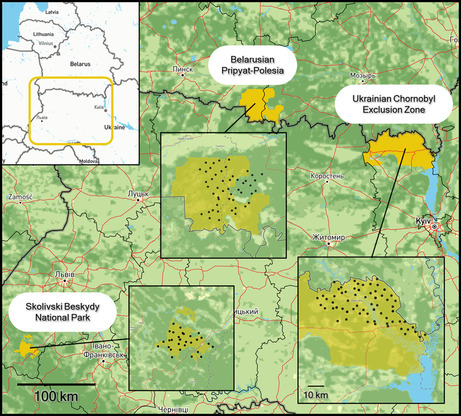

## Анотація

Рись євразійська (*Lynx lynx*) — велика хижа тварина, широко поширена в Євразії. Проте наше розуміння статусу популяції є різнорідним щодо їх поширення – деякі популяції ізольовані та страждають від генетичного дрейфу, а про деякі – немає жодної інформації. У багатьох європейських країнах моніторинг рисі євразійської здійснюється за допомогою демографічних досліджень, які мають вирішальне значення для її збереження та управління. Незважаючи на це, існують лише приблизні та фрагментовані оцінки популяцій в Україні та Білорусі, незважаючи на строгий захист в обох країнах та їх важливість для зв’язку рисі по всій Європі. Ми спостерігали за риссю з жовтня 2020 року по березень 2021 року та використовували фотопастки у поєднанні з методами «spatial capture‐recapture» (SCR; просторового захоплення‐перехоплення) за Баєсовим підходом, щоб надати першу оцінку щільності SCR трьох популяцій рисі в Україні та Білорусі, включно з українською Чорнобильською зоною відчуження, Південною Білорусю та Українськими Карпатами. Наші оцінки щільності варіювалися в межах досліджуваних територій від 0,45 до 1,54 особин/100 км2. Ця робота забезпечує суттєвий науковий компонент для загального розуміння збереження рисі в регіоні, де доступна лише загальна інформація, і відкриває двері для подальшого широкомасштабного моніторингу та оцінки тенденцій. Важлива інформація, яку ми надаємо, може значно покращити оцінку статусу цього охоронюваного виду в масштабах його поширення. Ми також обговорюємо наслідки для збереження рисі євразійської, незважаючи на геополітичні реалії, що впливають на моніторинг видів у регіоні. Наша робота є основою не тільки для майбутніх природоохоронних втручань, але й для оцінки наслідків порушень та загроз для цих популяцій, які охороняються.

## 1 INTRODUCTION

Large carnivores are keystone species with far‐reaching ecological effects on ecosystems (Wolf & Ripple, 2018) and have an important cultural and intrinsic value (Carlson et al., 2020). Despite legal protection in many countries, large carnivores are currently globally threatened by several anthropogenic influences such as habitat fragmentation, direct persecution and geopolitical unrest (Arlettaz et al., 2021; Heurich et al., 2018). Therefore, the status of all populations across the species’ range should be carefully assessed to establish conservation management plans. This information on large carnivores is crucially important, because in Europe's human‐dominated landscapes, large carnivores intersect human economic and social interests frequently, and detailed scientific evaluations allow evidence‐informed management and conservation interventions. The International Union for Conservation of Nature (IUCN) Red List of Threatened Species collects information indicating the global conservation status of large carnivores, which is determined through multiple risk categories. This is primarily done by assessing species range and abundances – the basic parameters for any population assessment (Breitenmoser‐Würsten et al., 2007). For the Eurasian lynx (*Lynx lynx*, hereafter “lynx”) in Europe, there are systematic approaches to status assessment and population monitoring (Weingarth et al., 2015; Zimmermann et al., 2013); however, these are not conducted evenly across the species range, leaving knowledge gaps for these indices.

Lynx are medium‐sized felids and are considered a key large carnivore in Europe. They are widely distributed across Eurasia (Breitenmoser & Breitenmoser‐Würsten, 2008; von Arx et al., 2021). In the European Union (EU), lynx are strictly protected under the Habitats Directive (Council Directive 92/43/EEC; EC, 1992), which aims to preserve important European biodiversity through connected conservation areas. As the Habitats Directive imposes obligations on monitoring designated species (Evans, 2012), such as lynx and wolves (*Canis lupus*), the status of large carnivore populations needs to be carefully monitored over time. EU countries share lynx populations across borders with Ukraine and Belarus, where lynx are protected under the Bern Convention (Council of Europe, 1979), ratified by Ukraine in 1999 and Belarus in 2013. Belarus denounced the treaty in August 2023. In Ukraine, lynx are also listed in the Red Data Book since 1994 and in Belarus since 1981. Although lynx are assigned to the IUCN risk category “Least Concern,” the status of populations is not homogeneous across their range (von Arx et al., 2021). Current population trends are decreasing in some cases, but the overall population trend is stagnant (von Arx et al., 2021). Smaller populations that remain isolated are at risk of reduced genetic variation caused by genetic drift, such as those reintroduced to central Europe (Mueller et al., 2022). Connectivity is therefore vital for lynx conservation in Europe (Bonn Lynx Expert Group, 2021; Premier et al., 2021).

In Central Europe, lynx have been systematically surveyed for the last decade to estimate abundance and density (Bonn Lynx Expert Group, 2021; Gimenez et al., 2019; Kubala et al., 2019), which form the basis of lynx conservation assessments and interventions, and further parameters such as apparent survival (Duľa et al., 2021; Palmero et al., 2021). However, several countries in Central and Eastern Europe, for example Ukraine and Belarus, do not fall under EU reporting obligations. Distribution and densities from these regions are ambiguous or fall below the standards of reporting set by the assessments conducted elsewhere (i.e. using shared, systematic monitoring methods), which can lead to conflicts (Kubala et al., 2021). This is highlighted in Cherepanyn et al. (2023), who point out the particular lack of common methodological approaches to lynx monitoring and the necessity to expand such in Ukraine. These countries are important for connectivity since lynx's dispersal ability allows animals in Ukraine and Belarus to readily move across borders, connecting populations in Central and Eastern Europe, for example Romania to Slovakia via the Ukrainian Carpathians linking the source with reintroduced population in Central/Western Europe. A harmonised monitoring across countries, despite differences in reporting obligations, is therefore inevitable and desirable for the conservation and management of lynx (Cherepanyn et al., 2023; Heurich et al., 2021), and recommendations have already been internationally defined (Boitani et al., 2015; Bonn Lynx Expert Group, 2021; Papp et al., 2020).

In Ukraine and Belarus, despite some protection, lynx were thought to be relatively rare due to poaching pressure and habitat degradation (Shkvyria & Shevchenko, 2009). However, recent general assessments of large carnivore numbers in Ukraine since 2009 indicated a stable number of lynx in the Carpathians and slightly positive trend in Polesia (Cherepanyn et al., 2023). Increases in areas around the Chornobyl Exclusion Zone in Ukraine and Belarus (Deryabina, 2008; Zhyla, 2002), situated in Polesia, have also been reported before 2009. Positive trends were noted in lynx populations over the last few decades in northern Belarus, where intensive monitoring is conducted (Sidorovich, 2022). Lynx in Belarus are most commonly yet imprecisely censused by local hunting communities, but efforts are not equally nor continuously undertaken in time and space, nor have they met scientific standards (Sidorovich, 2022). Similarly in Ukraine, lynx numbers are estimated alongside other game species by the State Forestry Agency from hunting units within their administrative areas (Cherepanyn et al., 2023). Similar efforts are made in protected areas. In Skolivski Beskydy National Park, an example from the Ukrainian Carpathians, lynx tracks are recorded across all park sectors, but without accounting for survey effort or using systematic recording schemes. In both Ukraine and Belarus, double counting over the borders of protected areas and forestry agencies is common (Zhyla, 2012). Without stronger statistical approaches, inferences about lynx numbers over wider areas are likely imprecise, which may lead to misunderstandings and conflicts with other land users, such as hunters (Kubala et al., 2021).

Camera traps are important tools for wildlife monitoring, especially for lynx due to their unique coat patterns, which allow the identification of individuals (Weingarth et al., 2012). In the Ukrainian Chornobyl Exclusion Zone, a recent study (Gashchak et al., 2022) used camera traps and non‐spatial capture–recapture methods to estimate a lynx population size of 53–68 individuals, with a relatively high density of 2.2–2.7 individuals/100 km^2^. However, non‐spatial methods are shown to overestimate the density of animals (Sollmann et al., 2011). Spatial capture–recapture (SCR) methods, explicitly incorporating fine‐scale spatial information associated with individual detections into population models, are a more popular (Tourani, 2022) approach to provide unbiased, more precise densities (Royle et al., 2014; Sollmann et al., 2011). To ensure statistical robustness, a proper field sampling design should be set with care regarding the spatial requirements of the target species (Sollmann et al., 2012) as opposed to random sampling often done with camera traps. The strength of this methodology is why it is utilised as a key component of lynx monitoring across Europe, for example Slovakia (Kubala et al., 2019), Germany and Czech Republic (Palmero et al., 2021) and Switzerland (Pesenti & Zimmermann, 2013).

We report here the first SCR density estimates of lynx from camera trapping in the Skolivski Beskydy National Park (SBNP) in the Ukrainian Carpathians, the Ukrainian Chornobyl Exclusion Zone (UCEZ), and three connected protected areas and a state forest in Belarusian Pripyat‐Polesia (BPP): Almany Mires Nature Reserve, Stary Zhaden Reserve, Topilla Bog and Bukchansky Forest. These are also the first lynx density estimations to the best of our knowledge from systematic work in the Ukrainian Carpathians from SBNP and the first for southern Belarus, from BPP. Since non‐spatial density estimates from camera traps are available for the UCEZ (Gashchak et al., 2022), our density estimates from SCR methods are a useful comparison. We expected our SCR densities to be lower than presented by the more recent, non‐spatial models. While the ecosystem types in BPP and the UCEZ differ from the mountainous SBNP, all areas have varying human disturbances. Accordingly, we expected to find varying densities across our study areas, with higher densities in UCEZ (strict human accessibility criteria) than SBNP or BPP (regular forestry, tourism, proximity to villages and roads).

## 2 MATERIALS AND METHODS

### 2.1 Study areas

**FIGURE 1:**
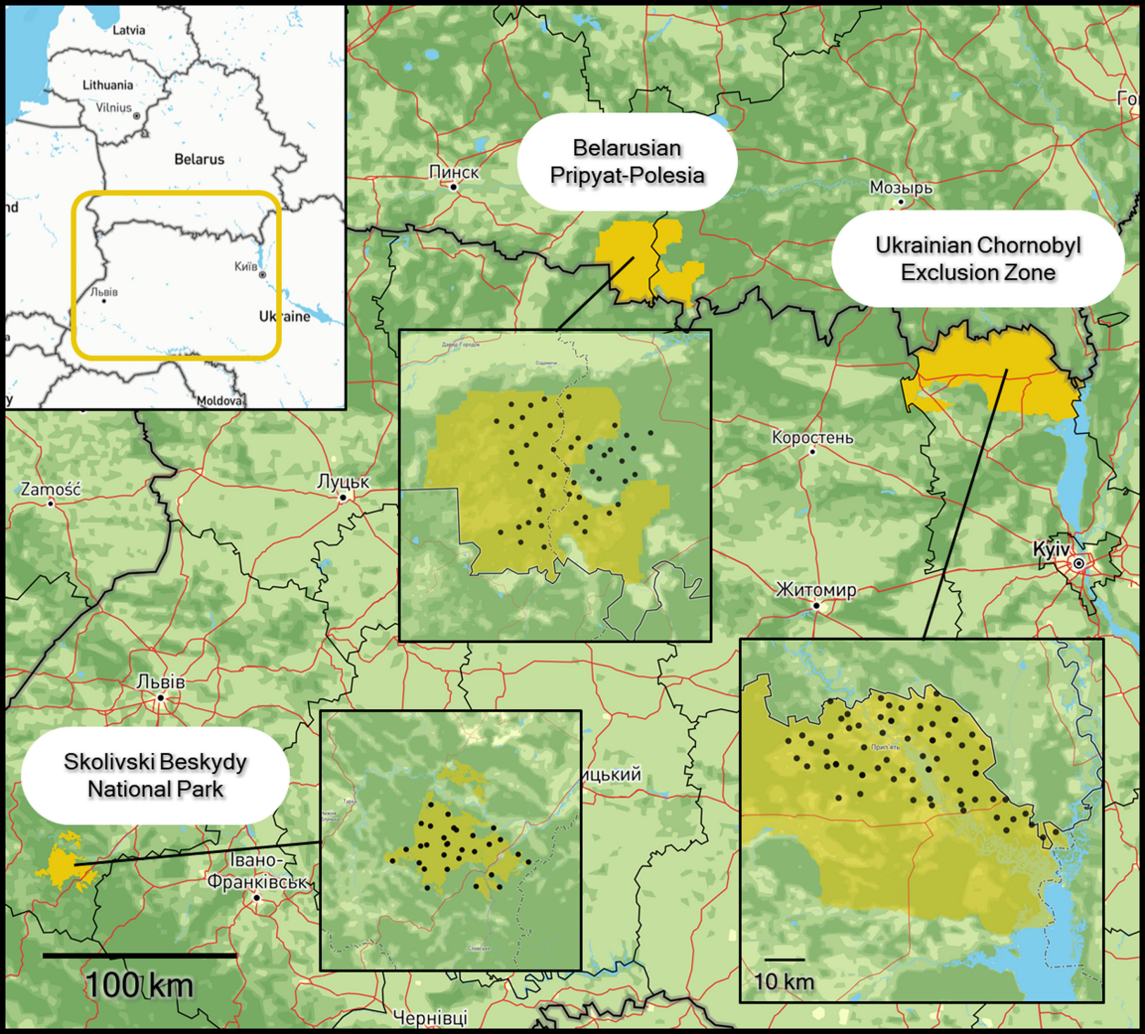
Map of the three study areas in which the lynx camera‐trapping monitoring was conducted. Protected areas where camera traps were placed are highlighted in yellow. Each black dot represents a paired camera‐trapping site.

We surveyed Eurasian lynx in two hotspots of European biodiversity: the Carpathian Mountains (western Ukraine) and Polesia (northern Ukraine and southern Belarus). They comprise distinct ecosystems: predominantly mixed‐mountain forests in the Carpathians and lowland forests, swamps, and mires in Polesia. Both boast large and important protected areas for wildlife. In the Ukrainian Carpathians, we surveyed lynx in Skolivski Beskydy National Park. In Polesia, we surveyed the Ukrainian Chornobyl Exclusion Zone and the protected area network of Belarusian Pripyat‐Polesia (Figure 1).

SBNP is located in the northeastern part of the Ukrainian Carpathian Mountains (49.1° N, 23.4E) in Lviv Oblast and is characterised by highly productive native fir (*Abies alba*) and beech (*Fagus sylvatica*) forests. The entire area comprises 353 km^2^ with a protected core zone of 52 km^2^. The absolute heights range from 600 to 1200 m.a.s.l., and the predominant landscape comprises of steeply sloping erosional wooded midlands shaped by denudation. The average height of the snow cover is about 40 cm during winter. The flora and fauna of the park are remarkably rich. Regarding mammals, European bison (*Bison bonasus*), red deer (*Cervus elaphus*), roe deer (*Capreolus capreolus*), wild boar (*Sus scrofa*), wolf (*Canis lupus*), brown bear (*Ursus arctos*) and European wildcat (*Felis silvestris*) are found here. The park is also a popular tourist destination, and the surrounding villages are associated with forestry, low‐intensity agriculture and recreation. Population density in Skolivski district is approximately 32 inhabitants/km^2^.

The UCEZ study area (2600 km^2^) is situated in Kyiv Oblast (51.4N, 30.1E) around the site of the most severe nuclear accident in history at the Chornobyl Nuclear Power Plant, in 1986 (Beresford et al., 2020; Kashparov et al., 2018). High levels of radioactive contamination led to the evacuation and relocation of the population over a large area. The topography is flat, lowland floodplain with old Scots pine (*Pinus sylvestris*) plantations, more natural deciduous forests and open areas of grassland following agricultural abandonment. The Pripyat River flows through the study area from north to south. The snow cover in winter is low with multiple annual melts. Apart from Eurasian lynx, the UCEZ counts red deer, roe deer, Przewalski's horse (*Equus przewalskii*), moose (*Alces alces*), European bison, wild boar, wolf and occasionally brown bear among the large mammals present. For almost 40 years since the nuclear accident in 1986, the UCEZ was effectively uninhabited except for guards, scientists, service staff and a handful of civilians concentrated in the town of Chornobyl (approximately 1000 inhabitants). The Chornobyl Radiation and Ecological Biosphere Reserve was established across 87% of the UCEZ, tasked with the preservation of biodiversity that has recovered after the accident.

BPP is located in the eastern part of Brest and the western part of Gomel, in southern Belarus along the Ukrainian border (51.7N, 27.4E). Small towns and villages border the western and northern parts of the study area, and the average population density of the surrounding districts is 12 inhabitants/km^2^. The largest protected area where monitoring took place was Almany Mires Nature Reserve. It is connected on the northeast to the protected area Stary Zhaden Reserve and on the southeast to Topilla Bog. On the east of Almany is the State Forest Bukchansky. Two further protected areas where monitoring did not occur are L'va Floodplain in the northwest and Pripyatsky National Park to the northeast. The entire landscape covers approximately 3500 km^2^. BPP comprises vast swamps and mires, with both planted coniferous and natural deciduous forests constituting Europe's largest complex of forests and swamps. In the study area, roe deer, moose, wild boar and wolf coincide with lynx. Forestry, illegal hunting and fishing, and fires are possible threats, and mushroom and berry picking are common seasonal pressures on the area.

### 2.2 Camera trapping

For reporting methods and results concerning the lynx monitoring, we followed the protocol from Palmero et al. (2023). The overall study period lasted from the end of October 2020 until March 2021. We used a 2.5 × 2.5 km grid for all study areas as previously used in the Carpathians by Kubala et al. (2019), applying a systematic design where one out of two cells was sampled (Zimmermann et al., 2013). We set camera traps in forests where landscape and terrain features increased the detection probability of lynx, particularly on forest roads, hiking trails, game paths and mountain ridges, or in locations based on previous signs of lynx presence (Blanc et al., 2013). Camera traps were stolen from three and two sites in SBNP and BPP, respectively, and at one site camera traps were destroyed in BPP. At each camera‐trapping site, we set two Xenon white flash camera traps (Cuddeback C‐series or G‐series) on opposite sides of the predicted lynx path to obtain high‐quality pictures of both flanks of animals. The camera traps were never placed exactly opposite each other to avoid mutual blinding from the flashes. Observers independently identified individual lynx in photographs based on their unique coat patterns. For SBNP, AFS identified the individuals, for BPP, AFS, Katharina Kasper and DS identified the individuals, and for UCEZ, the individuals were identified by SK and Maria Tryfonova. MG subsequently cross‐checked all pictures. If disparities arose, the individuals were discussed, but the final decision was taken by the most experienced observer (MG) (Choo et al., 2020; Young et al., 2019). Individuals were categorised into independent, juvenile and unknown when the individual was not identifiable (Weingarth et al., 2012). Juveniles, individuals <1‐year‐old, were considered as their mothers (Zimmermann et al., 2013), when this information was available, to increase the total number of recaptures. Independent individuals (>1‐year‐old) included subadult and adult lynx. Events with unidentifiable individuals were not considered further, but the numbers were reported. For some individuals in SBNP, only one‐flank pictures were available in this case. In particular, we collected three captures of three individuals for the left flank and five captures of three individuals for the right flank with one of them recaptured twice. These individuals were discarded to avoid overcounting, as one individual might be counted as two when the flanks are not matched. We collected sample sizes and the number of recaptures with the proportion of spatial ones. This helped us interpret the results as their precision generally increases with more recaptures (Palmero et al., 2023) and particularly with spread‐out spatial recaptures, as they inform the model about animal movement (Sollmann et al., 2012). These were calculated as the total number of different sites at which individuals were only recaptured. Additionally, we investigated the skewness of recaptures, that is if they are homogeneously distributed across individuals, as this positively influences the precision of results (Palmero et al., 2023; Sollmann et al., 2012).

### 2.3 Statistical analysis

We used a Bayesian SCR framework for density estimation, as Bayesian methods are shown to outperform the maximum likelihood estimator (MLE), especially when the sample size of individuals is low (Palmero et al., 2023; Royle et al., 2009), for example in SBNP. Sex was not available for most individuals from our study sites, as it was rarely visible from the photographs we collected. We could have fitted a model *M*_*h*_, but it would struggle to produce detection parameters for some individuals due to the reduced sample sizes resulting in overfitting and bias in the results. Therefore, we fitted a model *M*_*0*_, assuming a constant detection probability across individuals (Otis et al., 1978). Closed population density estimates were calculated using Markov Chain Monte Carlo (MCMC) algorithms in the R package “nimble” (de Valpine et al., 2017). We used data augmentation and set *M* for the theoretical population size to 100 for SBNP and BPP and 300 for the UCEZ, since the number of individuals detected was larger, ensuring convergence could be reached. This was checked through the Gelman–Rubin diagnostic statistics in the R package “coda” (Plummer et al., 2005) with 95% upper CI < 1.1 indicating convergence was reached. Convergence was also inspected visually. For all models, we run three chains by 10,000 iterations with a burn‐in of 2000.

The SCR methods rely on two detection parameters: the detection probability *g*_0_ and the detection function scale *σ*. The probability of detecting an individual is maximum at its theoretical activity centre, that is home range centre, and declines with distance from it. The activity centres of animals, including observed individuals and the theoretical population size *N*, are then distributed in the state space *S*, which is calculated as a square buffer around camera‐trapping sites of measure two to three times the detection function scale (Royle et al., 2014). When information on the home range size is available, this can be used to estimate sigma thus the buffer width. As this was not the case, we used the function “suggest.buffer” from the R package “secr” (Efford, 2022). This resulted in a recommended buffer width of 13, 16 and 20 km for SBNP, UCEZ and BPP, respectively.

To reduce temporal autocorrelation, we defined one occasion as 1 day and restricted the number of detections to at most one per site per occasion, following a Bernoulli distribution. Since we knew we would deal with reduced sample sizes, we tried to maximise the number of occasions for the analysis to maximise sample size and recaptures (Harmsen et al., 2020). Yet, we took care when overlapping the breeding season, as this can cause bias in the detection parameters (Dupont et al., 2019). Lynx start breeding in late February, with a peak in March (Göritz et al., 2006; Weingarth et al., 2015). Accordingly, we used data from November and December to February and excluded March as the peak of mating season (Table 1). Although it would have been safer to exclude February (when potential non‐resident lynx may arrive), we decided to include it because extending the survey length is a suitable compromise between improving the accuracy and precision of the results and violating demographic closure (Harmsen et al., 2020).

**TABLE 1:** The results of lynx camera‐trapping monitoring for the three study areas include spatial and temporal information on the sampling design and the total number of individuals and (spatial) recaptures with details on their skewness.

Study area	Sampling design						
	Size of the study area (minimum convex polygon) in km^2^	Number of camera‐trapping sites	Effective trapping nights	Number of one‐day occasions	Survey period	Total number of individuals and recaptures (spatial)	Number of individuals (recaptures)
Skolivski Beskydy National Park (SBNP)	390	35 (3)*	2730	90	Dec–Feb	5; 22 (10)	3 (5); 1 (4); 1 (3)
Ukrainian Chornobyl Exclusion Zone (UCEZ)	791	65 (0)*	6353	112	Nov–Feb	22; 24 (19)	1 (8): 1 (7); 1 (3); 6 (1); 13 (0)
Belarusian Pripyat‐Polesia (BPP)	811	50 (2)*	5750	120	Nov–Feb	14; 65 (13)	1 (18); 1 (12); 2 (9); 1 (8); 1 (5); 1 (2); 2 (1); 5 (0)

*Note*: Asterisk (*) denotes sites with stolen or broken camera traps.

Bayesian density estimates are reported with point estimates and highest posterior density (HPD) intervals. We also calculated the precision of density estimates using the coefficient of variation (CV), which was obtained as the ratio between the posterior standard deviation and the posterior mean of realised densities. A CV of 0.20 is associated with high precision (Efford & Boulanger, 2019).

As a last step, we used the posterior distribution of activity centres of realised individuals (Table 1) to calculate the density for the minimum convex polygon (MCP). Specifically, we divided the number of all realised activity centres from all iterations by the total number of iterations (30,000); hence, we divided this number by the area of the MCP and obtained its specific lynx density. We then created a raster with the activity centres in the study area. This allowed us to visualise animal distribution in space and make inferences about habitat use.

## 3 RESULTS

### 3.1 Camera trapping

BPP had the highest number of occasions and recaptures, although these were not equally distributed across individuals (14) and had the smallest proportion of spatial recaptures (13 out of 65). The UCEZ had the largest sample size of individuals (22), the highest proportion of spatial recaptures (19 out of 24) and the highest number of camera‐trapping sites. SBNP had the lowest sample size of individuals (5) but a moderate proportion of spatial recaptures (10 out of 22). The skewness of recaptures was generally poor (Table 1; Appendix S1). We had 10, 0 and 7 unidentified lynx events in SBNP, UCEZ and BPP, respectively (e.g. overexposed images, coat patterns not visible).

### 3.2 Statistical analysis

All Gelman–Rubin diagnostic statistics for the three models had a 95% upper CI < 1.1 for all parameters indicating convergence (Appendix S2). This was also inspected visually (Appendix S3).

**FIGURE 2:**
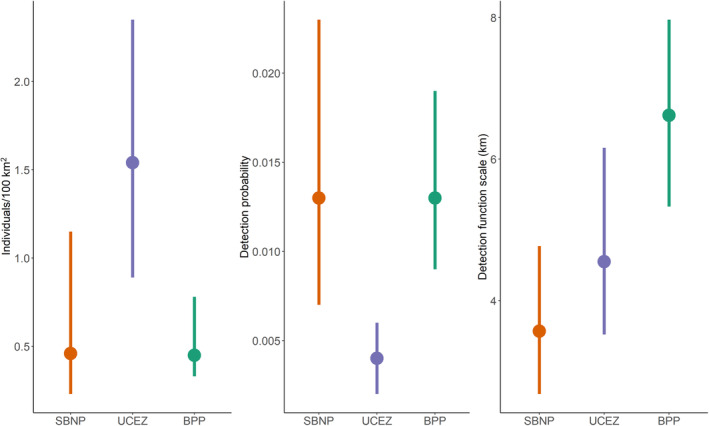
Results of the SCR analysis with density estimates, detection function scale and detection probability for the three study areas. Bars refer to the Bayesian HPD intervals.

Densities varied across study areas. The highest values were observed in the UCEZ, with 1.54 individuals/100 km^2^ (HPD intervals 0.89–2.35), and the lowest in BPP with 0.45 individuals/100 km^2^ (0.33–0.78), respectively (Figure 2). In SBNP, the densities recorded were 0.46 individuals/100 km^2^ (0.23–1.15; Figure 2). UCEZ and BPP density estimates had moderate precision (CV = 0.23 for both areas), while the CV was higher for SBNP (0.38). The detection probability was generally low, particularly in the UCEZ, while the detection function scale was largest in BPP (Figure 2).

**FIGURE 3:**
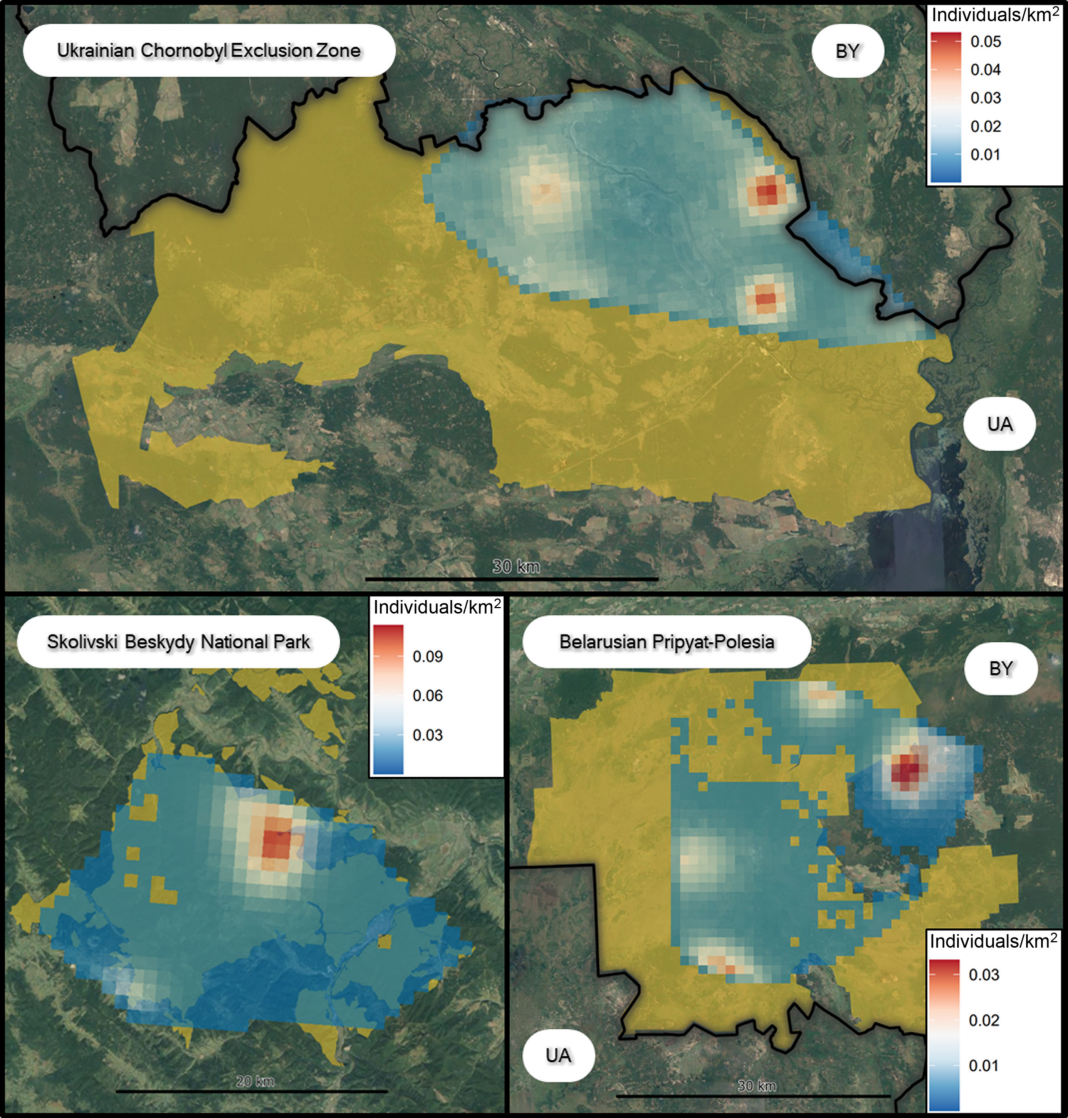
Raster map of the posterior distribution of activity centres for the realised individuals within the MCP of the camera‐trapping array for each study area. Raster pixels have an area of 1 km^2^, and lynx density is expressed in individuals/km^2^. The highlighted area represents the protected portion of each study area.

Densities for the MCPs were 0.81, 0.96 and 0.31 for SBNP, the UCEZ and BPP, respectively. The distribution of activity centre clusters was patchy in all study areas, especially in BPP with a central wide band of no density, and these were always located at the edges of MCP boundaries, except for one cluster in the UCEZ (Figure 3).

## 4 DISCUSSION

We used camera trapping and SCR models in a Bayesian framework to provide the first SCR density estimates for three lynx populations in Ukraine (Polesia and the Carpathians) and Belarus (Polesia). By providing the first spatially explicit densities for lynx in Ukraine and Belarus, our work carries important conservation messages for the species in these countries and the rest of Europe. Firstly and most importantly, we followed the recommendations of multiple international groups on the monitoring of lynx for conservation (Boitani et al., 2015; Bonn Lynx Expert Group, 2021; Kubala et al., 2021) on the implementation of systematic monitoring methods in two European countries where this was not accomplished before. This gives us the most valuable and comparable lynx density estimates for Ukraine and Belarus to date. Although concrete distribution and connectivity maps are still missing, building on work by Cherepanyn et al. (2023), we add another piece of optimistic evidence concerning the reportedly negative lynx status in the Ukrainian Carpathians noted by the (Bonn Lynx Expert Group, 2021).

Our closed population density estimates ranging from 0.45 to 1.54 individuals/100 km^2^ are similar to those from other Central European lynx populations estimated using comparable methods. Lynx density ranged from 0.24 to 0.91 individuals/100 km^2^ in the French Jura Mountains (Gimenez et al., 2019). In two areas of the Swiss Alps, lynx density was 1.38 and 1.47 individuals/100 km^2^ (Pesenti & Zimmermann, 2013). In the Bohemian Forest Ecosystem, lynx density was estimated using open population models ranging from 0.69 to 1.33 individuals/100 km^2^ (Palmero et al., 2021). Lynx densities from the Carpathians Mountains in other countries were also comparable: 0.26–1.85 individuals/100 km^2^ of suitable habitat in the Western Carpathians (Duľa et al., 2021), 0.58 and 0.81 individuals/100 km^2^ in two areas of the Slovak Carpathians (Kubala et al., 2019) and 1.60–1.70 individuals/100 km^2^ in the Romanian Carpathians (Iosif et al., 2022). The only densities higher than these were observed in Turkey: 4.20 individuals/100 km^2^ (Avgan et al., 2014), where surveyed lynx, however, belong to a different subspecies (Caucasian lynx; *Lynx lynx dinniki*) mainly feeding on lagomorphs and thus having smaller home ranges. All the abovementioned studies were instead conducted on the Carpathian lynx (*Lynx lynx carpathicus*). Our results indicate the first indication that the densities of lynx in our study sites are comparable to other regions in Central and Eastern Europe. This is also considering nearly all the aforementioned studies are multi‐season surveys with well‐established sampling designs. Our single‐season surveys are also a limitation, however. Lynx population fluctuations in both Carpathian (Duľa et al., 2021) and European lynx populations overall (Gimenez et al., 2019; Palmero et al., 2021) are well established. However, the threats to lynx in our study sites are not well defined. Our expectation is that lynx in the UCEZ are the least threatened due to its size and inaccessibility, while both SBNP and BPP are more at risk of illegal killings, fragmented connectivity and quality, and lower prey availabilities, and this is reflected in the lower densities we report. Legal or illegal hunting of lynx prey species, such as roe deer, could lower the prey availability for large carnivores, and these areas had relatively lower numbers of camera trap observations of prey species than UCEZ. Also, Kubala et al. (2021)) point out that illegal killing may result in significant mortality in the Slovakian lynx population, despite being as yet untested, as was shown in Czechia (Červený et al., 2019) in Poland (Kowalczyk et al., 2015). We have little reason to expect Ukraine or Belarus would be different in this regard.

Despite frequent pressures and threats, protected areas likely play a significant part in the conservation of lynx in Ukraine and Belarus. Only in BPP were a significant number of camera traps outside of protected areas, and as such, our results should be viewed from a protected areas context. The interplay between protected and unprotected areas for lynx in these regions is not fully understood, but protected areas can host scientific monitoring, especially if the necessary support and funding for monitoring programmes are established (Cherepanyn et al., 2023). Protected area boundaries are not frontiers to the distributions of large carnivores in Europe (Cimatti et al., 2021; Terraube et al., 2020) and are often insufficient for effectively protecting large carnivores (Diserens et al., 2017). However, they provide key refugia and important islands of protection in fragmented, dangerous landscapes (Müller et al., 2014; Smith et al., 2022). Each protected area where we conducted monitoring adjoins other areas with landscape protection, ensuring some degree of connectivity is possible. However, the question of protected area effectiveness is still immediate, as illegal killing similar to other areas of Europe could still be a factor (Červený et al., 2019; Heurich et al., 2018).

Suitable habitat for lynx in Europe's fragmented landscapes is constrained by human disturbance, which lynx avoid (Oeser et al., 2023; Ripari et al., 2022). Reintroduced lynx populations in Central Europe also exhibit genome‐wide diversity loss, requiring the immediate need to increase connectivity between populations (Mueller et al., 2022; Papp et al., 2020; Premier et al., 2021). However, the extent to which Ukrainian and/or Belarusian lynx populations suffer from the same pressures regarding connectivity and genetics is unknown, limiting our knowledge until further research is replicated in these regions (Papp et al., 2020). Building on our study of lynx densities, the effective conservation of lynx in Polesia and the Ukrainian Carpathians will require understanding the distribution and habitat associations (e.g. occupancy, Van der Weyde et al., 2022) as well as genetic health (Mueller et al., 2022). This combination of recommendations, especially using genetics and camera trapping, is already reflected in the recommendations by Boitani et al. (2015), Bonn Lynx Expert Group (2021) and Papp et al. (2020) and should guide successful conservation interventions for the continued conservation of lynx in Ukraine, Belarus and across Europe.

The precision of our density estimations increased with individual sample size and the number of recaptures, following the other studies (Palmero et al., 2023; Sollmann et al., 2012). Our most precise density estimates were in the UCEZ, with the largest sample size and the highest proportion of spatial recaptures. On the contrary, we observed low precision in the area (SBNP) with the smallest sample size, but a reasonable number of (spatially distributed) recaptures (~50%). This is in line with Palmero et al. (2023) showing the sample size of individuals as the overall most important variable influencing the precision of SCR density estimates. Here, sample size was reduced because we excluded single flanks from the dataset, potentially losing six individuals. Low precision for this study area was expected as the number of camera traps was limited and the national park boundaries are too small to encompass enough individuals, that is >10 that are recaptured at least twice for moderate precision and >20 that are recaptured at least once for high precision (Palmero et al., 2023). The number of occasions in SBNP was slightly lower due to a shorter monitoring period starting in December because of logistical constraints reducing recaptures. We did not have a priori information on area‐specific home range sizes. However, based on estimates from nearby areas, that is, 165 km^2^ for males in the Białowieża Forest (Schmidt et al., 1997), the size of the MCP for SBNP (353 km^2^) covered between two and three male home ranges. For improving the sampling design in the future, we suggest enlarging the MCP through cooperation with other landowners and users and redefining the spacing of camera traps based on relevant telemetry data. To reduce the number of overexposed images, camera trap locations should be tested and flash intensity reduced if possible. In BPP and the UCEZ, the size of the MCP was almost five times larger than a male home range, ensuring larger sample sizes thus more precise results would be obtained (Efford & Boulanger, 2019; Tobler & Powell, 2013).

Apart from the limitations in SBNP, all areas were affected by poor skewness of the recaptures, potentially decreasing the precision (Palmero et al., 2023; Sollmann et al., 2012). This was probably due to low detection probabilities (<0.015 in all areas). In the UCEZ, this parameter was particularly low because 13 out of 22 individuals were only captured once. The sampling design we used ensured that at least one camera‐trapping site was set in all lynx home ranges, but this was probably insufficient to provide enough (spatial) recaptures, with multiple sites within the smallest home range of the target species (Sollmann et al., 2012; Tobler & Powell, 2013). On the other hand, using data from February potentially included resident males making excursions during the mating seasons in search of a partner. These individuals generally decrease overall capture probabilities because they have no established home ranges and inflate density estimates (Larrucea et al., 2007). However, the benefits of extending the survey length to increase sample size and recaptures are shown to overcome the potential risk of violating demographic closure caused by immigration, emigration, recruitment and mortality (Harmsen et al., 2020). This may explain the relatively high precision for density estimates in the UCEZ where our increased sample size and recaptures overcame the low detection probability.

In the UCEZ, the highest activity centre densities in our monitoring area were located in two patches, east of the Pripyat River and another west of Chornobyl town at the crossroads of several forested areas. In BPP, the lowest activity centre density fell over an area of acute human disturbance, where a large forest road north‐to‐south facilitates forestry and trucks, tractors, cars, motorcycles and other human activities. BPP had the largest MCP size with many camera‐trapping sites, yet density was relatively low. Here, an extensive proportion of the area is composed of mires and marshlands unsuitable for lynx when waterlogged. This may explain the observed low density and the large detection function scale, as lynx have to travel longer linear distances over dryer patches to access resources. Densities from the state spaces were higher than the MCPs in BPP (0.45 vs 0.31) and the UCEZ (1.54 vs 0.96) but lower for SBNP (0.46 vs 0.80).

However, we could not calculate the uncertainty for the estimates from the MCP and since these estimates always fell within the HPD intervals of the original estimates, little can be discussed. However, we offer a short explanation for the lower values in UCEZ and BPP. Both BPP and the UCEZ are extensive landscapes connected to other suitable and protected lynx areas across country borders, without “hard borders” in the arrays. Therefore, the exact placement of our arrays in the wider landscape may mean the MCPs did not always enclose the most probable areas to detect lynx because the wetlands, rivers and landscape heterogeneity within the array may push the activity centre clusters away from the centre of arrays. To overcome these aspects in future surveys, we recommend using habitat suitability maps (Oeser et al., 2023), simulating different sampling designs (Ash et al., 2020) or employing pre‐season surveys to inform camera trap placements.

Lynx density in the UCEZ was recently estimated using non‐spatial capture–recapture models and mean maximum distance moved (MMDM) (Gashchak et al., 2022). High abundance and density were attributed to factors such as suitable habitat, absence of human activities and an abundant prey base. However, the sampling designs used in these projects were not tailored to lynx and were inconsistent across the study areas. Additionally, some study areas were particularly small (20–175 km^2^). Density estimates from non‐spatial methods are often inflated because of underestimated MMDM, especially for small study areas (Sollmann et al., 2011; Whittington & Sawaya, 2015; Zimmermann et al., 2013). Therefore, future demographic studies should prioritise SCR estimates to establish effective monitoring plans.

When species have sex‐specific detection probability and detection function scale, such information should be included in the model to avoid bias. Otherwise, density would decrease as a result of a sample dominated by individuals with a higher detection probability and detection function scale (Sollmann et al., 2011). However, sex can only be determined if a female is detected with kittens or genital parts are photographed (Weingarth et al., 2012). This information is often missing for most individuals, especially in previously unsurveyed areas where they have not been camera‐trapped over time. We could not account for sex in the model resulting in potentially underestimated densities. To solve this issue in the future, a long‐term monitoring plan should endeavour to track individuals over time (life histories) and allow sex determination to improve estimates.

The need for harmonised, scientific monitoring of Eurasian lynx is well established (Boitani et al., 2015; Bonn Lynx Expert Group, 2021), and there are huge opportunities to enhance assessments of lynx status where data gaps exist (Kubala et al., 2021). For example, gathering data collected outside of research and in Ukrainian language, as demonstrated in (Cherepanyn et al., 2023). Ukraine is also a candidate member for the EU, and a future ascension would presumably invoke standardised reporting of lynx monitoring under the Habitats Directive (EC, 1992). Unfortunately, current geopolitical realities reflect that international collaboration in the monitoring and management of lynx between other European nations and Belarus is now halted. Belarus also denounced the Bern Convention, under which lynx are protected, in August 2023. Concurrently, in Ukraine, it has been severely limited. In February 2022, soon after our study was conducted, the Russian Federation commenced a full‐scale invasion of Ukrainian sovereign territory (including our study area in the UCEZ) from both Russian and Belarusian borders. This directly risks protected landscapes and wildlife populations, jeopardises the establishment of long‐term monitoring of Eurasian lynx in both Polesia and the Ukrainian Carpathians, and affects academic research (Orizaola et al., 2022). While we do not assess the impacts of war on wildlife in this study, it must be noted that the continuity of lynx research and collaboration in the Polesia region, between Ukraine and Belarus, has been stopped. Not only do our results provide the first spatially explicit density estimates for important regions in Polesia, they now provide the only baseline densities before the Russian invasion of Ukraine, which may allow insights into how the war affects this protected species.

## AUTHOR CONTRIBUTIONS

**Stefano Palmero:** Conceptualization (equal); formal analysis (equal); methodology (equal); writing – original draft (lead); writing – review and editing (lead). **Adam F. Smith:** Conceptualization (equal); data curation (equal); resources (equal); writing – original draft (lead); writing – review and editing (lead). **Svitlana Kudrenko:** Conceptualization (equal); data curation (equal); project administration (equal); writing – review and editing (equal). **Martin Gahbauer:** Conceptualization (equal); data curation (equal). **Dominik Dachs:** Formal analysis (equal); methodology (equal); resources (equal); software (lead). **Kirsten Weingarth‐Dachs:** Formal analysis (equal); methodology (equal); resources (equal); software (lead). **Irina Kashpei:** Data curation (equal); project administration (equal). **Dmitry Shamovich:** Data curation (equal); resources (equal). **Denys Vyshnevskiy:** Project administration (equal); resources (equal). **Oleksandr Borsuk:** Resources (equal). **Kateryna Korepanova:** Resources (equal). **Andriy‐Taras Bashta:** Resources (equal). **Rostyslav Zhuravchak:** Project administration (equal). **Viktar Fenchuk:** Conceptualization (equal); project administration (equal). **Marco Heurich:** Conceptualization (equal); project administration (lead); resources (equal); supervision (equal); writing – review and editing (equal).

## CONFLICT OF INTEREST STATEMENT

The authors have no competing interests to be declared.

## Supporting information


Appendix S1–S3

## Data Availability

Data and code used for the analysis can be downloaded at the following Dryad link: DOI: 10.5061/dryad.rbnzs7hhx. The private link for the peer review is the following: https://datadryad.org/stash/share/lezmbodW8BuOzqr_KqOgu9zqxL1n4Yb0ti1hhVLMqwU.
