# Supplementary material for: Shining a light on elusive lynx: Density estimation of three Eurasian lynx populations in Ukraine and Belarus
Source: Ecol Evol. 2023 Nov 9;13(11):e10688. doi: 10.1002/ece3.10688 (PMC10636425; doi:10.1002/ece3.10688)
Supplement: Supplementary file 1 — Appendix S1–S3 [file ECE3-13-e10688-s001.docx]

**Appendix**

| Individual ID | Occasion | | | | | | | | | | | | | | | | | | | | | | | | | | | | | | | | | | | | | | | | | | | | | | | | | | | | | | | | | | | | |  |
| --- | --- | --- | --- | --- | --- | --- | --- | --- | --- | --- | --- | --- | --- | --- | --- | --- | --- | --- | --- | --- | --- | --- | --- | --- | --- | --- | --- | --- | --- | --- | --- | --- | --- | --- | --- | --- | --- | --- | --- | --- | --- | --- | --- | --- | --- | --- | --- | --- | --- | --- | --- | --- | --- | --- | --- | --- | --- | --- | --- | --- | --- | --- |
|  | 1 | 2 | 3 | 4 | 5 | 6 | 7 | 8 | 9 | # | # | # | # | # | # | # | # | # | # | # | # | # | # | # | # | # | # | # | # | # | # | # | # | # | # | # | # | # | # | # | # | # | # | # | # | # | # | # | # | # | # | # | # | # | # | # | # | # | # | # | # | ## # # # # # # # # # # # # # # # # # # # # # # # # # # # |
| B1 |  | • | • |  |  |  | • |  |  |  |  |  |  |  |  |  |  |  |  |  |  |  |  |  |  |  |  |  |  |  |  |  |  |  |  |  |  |  |  |  |  | • |  |  |  |  |  |  |  |  | • |  |  |  |  |  |  |  |  |  |  |  |
| B2 |  |  |  |  |  |  |  |  |  |  |  |  |  |  |  |  |  |  |  |  |  |  |  |  |  |  |  |  |  |  |  |  |  |  |  |  |  |  |  |  |  |  |  |  |  |  |  |  |  |  |  |  |  |  |  |  |  | • |  |  |  | • • • • |
| B3 |  |  |  |  |  | • |  |  |  |  |  |  |  |  |  |  |  |  |  |  | • |  |  |  |  |  |  |  |  |  |  |  |  |  | • |  | • |  | • |  |  |  |  |  |  |  |  |  |  |  |  |  |  |  |  |  |  |  |  |  |  | • |
| B4 |  |  |  |  |  |  |  |  |  |  |  |  |  |  |  |  |  |  |  |  |  |  |  |  |  |  |  |  |  |  |  |  |  |  |  |  | • |  |  |  |  |  |  |  |  |  |  |  |  |  |  |  |  |  |  |  |  |  |  |  |  | • • • |
| B5 |  |  |  |  | • |  |  |  |  | • |  |  |  |  |  |  |  | • |  |  |  |  |  |  |  |  |  |  |  |  |  |  |  |  |  |  | • |  |  | • |  |  |  |  |  |  |  |  |  |  |  |  |  |  |  |  |  |  |  |  |  | • |

**Appendix S1a.** Capture histories of all individuals detected in the SBNP.

| Individual ID | Occasion | | | | | | | | | | | | | | | | | | | | | | | | | | | | | | | | | | | | | | | | | | | | | | | | | | | | | | | | | | | | |  |
| --- | --- | --- | --- | --- | --- | --- | --- | --- | --- | --- | --- | --- | --- | --- | --- | --- | --- | --- | --- | --- | --- | --- | --- | --- | --- | --- | --- | --- | --- | --- | --- | --- | --- | --- | --- | --- | --- | --- | --- | --- | --- | --- | --- | --- | --- | --- | --- | --- | --- | --- | --- | --- | --- | --- | --- | --- | --- | --- | --- | --- | --- | --- |
|  | 1 | 2 | 3 | 4 | 5 | 6 | 7 | 8 | 9 | # | # | # | # | # | # | # | # | # | # | # | # | # | # | # | # | # | # | # | # | # | # | # | # | # | # | # | # | # | # | # | # | # | # | # | # | # | # | # | # | # | # | # | # | # | # | # | # | # | # | # | # | ## # # # # # # # # # # # # # # # # # # # # # # # # # # # # # # # # # # # # # # # # # # # # # # # # # |
| B1 |  |  |  |  |  |  |  |  |  |  |  |  |  |  |  |  |  |  |  |  |  |  |  |  |  |  | • |  |  |  |  |  |  |  |  |  |  |  |  |  |  |  |  |  |  |  |  |  | • |  |  |  |  |  |  |  |  |  |  |  |  |  |
| B10 |  |  |  |  |  |  |  |  |  |  |  |  |  |  |  |  |  |  |  |  |  |  |  |  |  |  |  |  |  |  |  |  |  |  |  |  |  |  |  |  |  |  |  | • |  |  |  |  |  |  |  |  |  |  |  |  |  |  |  |  |  |  |
| B11 |  |  |  |  |  |  |  |  |  |  |  |  |  |  |  |  |  |  |  |  |  |  |  |  |  |  |  |  |  |  |  |  |  |  |  |  |  |  |  | • |  |  |  |  |  |  |  |  |  |  |  |  |  |  |  |  |  |  |  |  |  | • |
| B12 |  |  |  |  |  |  |  |  |  |  |  |  |  |  |  |  |  |  |  |  |  |  |  |  |  |  |  |  |  |  |  |  |  |  |  |  |  |  |  |  |  |  |  |  |  |  |  |  |  |  |  |  |  |  |  |  |  |  | • |  |  |  |
| B13 |  |  |  |  |  |  |  |  |  |  |  |  |  |  |  |  |  |  |  |  |  |  |  |  |  |  |  |  |  |  |  |  |  |  |  |  |  |  |  |  |  |  | • |  |  |  |  |  |  |  |  |  |  |  |  |  |  |  |  |  |  |  |
| B14 |  |  |  |  |  |  |  |  |  |  |  |  |  |  |  |  |  |  |  |  |  |  |  |  |  |  |  |  |  |  |  |  |  |  |  |  |  |  |  |  |  |  |  |  |  |  |  |  |  |  |  |  |  |  |  |  |  |  |  |  |  | • |
| B16 |  |  |  |  |  |  |  |  |  |  |  |  |  |  |  |  |  |  |  |  |  |  |  |  |  |  |  |  |  |  |  |  |  |  |  |  |  |  |  |  |  |  |  |  |  |  | • |  |  |  |  |  |  |  |  |  |  |  |  |  |  |  |
| B17 |  |  |  |  |  |  |  |  |  |  |  |  |  |  |  |  |  |  |  |  |  |  |  |  |  |  |  |  |  |  |  |  |  |  |  |  |  |  |  | • |  |  |  |  |  |  |  |  |  |  |  |  |  |  |  |  |  |  |  |  |  |  |
| B18 |  |  |  |  |  |  |  |  |  |  |  |  |  |  |  |  |  |  |  |  |  |  |  |  |  |  |  |  |  |  |  |  |  |  |  |  |  |  |  |  |  |  |  | • |  |  |  | • |  |  | • |  |  |  |  |  |  | • |  |  |  |  |
| B19 |  |  |  |  |  |  |  |  |  |  |  |  |  |  |  |  |  |  |  |  |  |  |  |  |  |  |  |  |  |  |  |  |  |  |  |  |  |  |  |  |  |  |  |  |  |  |  |  |  |  |  |  |  |  |  |  |  |  |  |  |  | • • |
| B2 |  |  |  |  |  |  |  |  |  |  |  |  |  |  |  |  |  |  |  |  |  |  |  |  |  |  |  |  |  |  |  |  |  |  |  |  |  |  |  |  |  |  |  |  |  |  |  |  |  |  |  |  |  |  |  |  |  |  |  |  |  | • |
| B20 |  |  |  |  |  |  |  |  |  |  |  |  |  |  |  |  |  |  |  |  |  |  |  |  |  |  |  |  |  |  |  |  | • |  |  |  |  |  |  |  |  |  |  |  |  |  |  |  |  |  |  |  |  |  |  |  |  |  |  |  |  |  |
| B21 |  |  |  |  |  |  |  |  |  |  |  |  |  |  |  |  |  |  |  |  |  |  |  |  |  |  |  |  |  |  |  |  |  |  |  |  |  |  |  |  |  |  |  |  |  |  |  |  |  |  |  |  |  |  |  |  |  |  |  |  |  | • |
| B22 |  |  |  |  |  |  |  |  |  |  |  |  |  |  |  |  |  |  |  |  |  |  |  |  |  | • |  |  |  |  |  |  |  |  |  |  |  |  |  |  |  |  |  |  |  |  |  |  |  |  |  |  |  |  |  |  |  |  |  |  |  | • |
| B23 |  |  |  |  |  |  |  |  |  |  |  |  |  |  |  |  |  |  |  |  |  |  |  |  |  |  |  |  |  |  |  |  |  |  |  |  |  |  |  |  |  |  |  |  |  |  |  |  |  |  |  |  |  |  |  |  |  |  |  |  |  | • |
| B24 |  |  |  |  |  |  |  |  |  |  |  |  |  |  |  |  |  |  |  |  |  |  |  |  |  |  |  |  |  | • |  |  |  |  |  |  |  |  |  |  |  |  |  |  |  |  |  |  |  |  |  |  |  |  |  |  |  |  |  |  |  |  |
| B3 |  |  |  |  |  |  |  |  |  |  |  |  |  |  |  |  |  |  |  |  |  |  |  |  |  |  |  |  |  |  | • |  |  |  |  |  |  |  |  |  |  |  |  |  |  |  |  |  |  |  |  |  |  |  |  |  |  |  |  |  |  | • |
| B5 |  |  |  |  |  |  |  | • |  |  | • |  |  |  |  |  |  |  |  |  | • |  |  |  |  |  |  |  |  | • |  |  |  |  |  |  |  |  |  |  |  |  |  |  |  |  |  |  |  |  |  |  |  |  |  |  |  |  |  | • |  | • • • • |
| B6 |  |  |  |  |  |  |  |  |  |  |  |  |  |  |  |  |  |  |  |  | • |  |  |  |  |  |  |  |  |  |  |  |  |  |  |  |  |  |  |  |  |  |  |  |  |  |  |  |  |  |  |  |  |  | • |  |  |  |  |  |  | • • • • • |
| B7 |  |  |  |  |  |  |  |  |  |  |  |  |  |  |  |  |  |  |  |  |  |  |  |  | • |  |  |  |  |  |  |  |  |  |  |  |  |  |  |  |  |  |  |  |  |  |  |  |  |  |  |  |  |  |  |  |  |  |  |  |  | • |
| B8 |  |  |  |  |  |  |  |  |  |  |  |  |  |  |  |  |  |  |  |  |  |  |  |  |  |  |  |  |  |  |  |  |  |  |  |  |  |  |  |  |  |  |  |  |  |  |  |  |  |  |  |  |  |  |  |  |  |  |  |  |  | • |
| B9 |  |  |  |  |  |  |  |  |  |  |  |  |  |  |  | • |  |  |  |  |  |  |  |  |  |  |  |  |  |  |  |  |  |  |  |  |  |  |  |  |  |  |  |  |  |  |  |  |  |  |  |  |  |  |  |  |  |  |  |  |  |  |

**Appendix S1b.** Capture histories of all individuals detected in the UCEZ.

| Individual ID | Occasion | | | | | | | | | | | | | | | | | | | | | | | | | | | | | | | | | | | | | | | | | | | | | | | | | | | | | | | | | | | | |  |
| --- | --- | --- | --- | --- | --- | --- | --- | --- | --- | --- | --- | --- | --- | --- | --- | --- | --- | --- | --- | --- | --- | --- | --- | --- | --- | --- | --- | --- | --- | --- | --- | --- | --- | --- | --- | --- | --- | --- | --- | --- | --- | --- | --- | --- | --- | --- | --- | --- | --- | --- | --- | --- | --- | --- | --- | --- | --- | --- | --- | --- | --- | --- |
|  | 1 | 2 | 3 | 4 | 5 | 6 | 7 | 8 | 9 | # | # | # | # | # | # | # | # | # | # | # | # | # | # | # | # | # | # | # | # | # | # | # | # | # | # | # | # | # | # | # | # | # | # | # | # | # | # | # | # | # | # | # | # | # | # | # | # | # | # | # | # | ## # # # # # # # # # # # # # # # # # # # # # # # # # # # # # # # # # # # # # # # # # # # # # # # # # # # # # # # # # |
| B1 |  |  |  | • |  |  |  |  |  |  |  |  |  |  |  |  |  |  | • |  |  |  |  |  |  |  |  |  |  |  |  |  |  |  |  |  | • | • |  |  |  |  |  |  |  |  |  |  |  | • |  |  |  |  |  |  |  |  |  |  |  | • |
| B10 |  |  |  |  |  |  |  |  |  |  |  |  |  |  |  |  |  |  |  |  |  |  |  |  |  |  |  |  |  |  |  |  |  |  |  |  |  |  |  |  |  |  |  |  |  |  |  |  | • |  |  |  |  |  |  |  |  |  |  |  |  |  |
| B2 |  |  |  |  |  |  |  |  |  |  |  |  |  |  |  |  |  |  |  |  |  |  |  |  |  |  |  |  |  |  |  |  |  |  |  |  |  |  |  |  |  |  | • |  |  |  |  |  |  |  |  |  |  |  |  |  |  |  |  |  |  |  |
| B3 |  |  |  |  |  |  |  |  | • |  |  |  |  |  |  |  |  |  |  | • |  |  |  |  |  |  |  |  |  |  |  |  |  |  |  |  |  |  |  |  |  |  |  |  |  |  |  |  |  |  |  |  |  |  |  |  |  |  |  |  |  | • |
| B4 |  |  |  |  | • | • |  |  |  |  | • |  |  |  |  |  |  |  |  |  |  |  |  |  |  | • |  |  |  |  |  |  |  |  |  |  |  |  |  |  |  |  |  |  |  |  |  |  |  |  |  |  | • |  |  |  |  |  | • |  |  | • • • • • |
| B6 |  |  |  |  |  |  |  |  |  |  |  |  |  |  |  |  |  |  |  |  |  |  |  |  |  |  |  |  |  |  |  | • |  |  |  |  |  |  |  |  |  | • | • |  |  |  |  | • |  |  |  |  |  |  |  |  |  |  |  |  |  |  |
| B7 |  |  |  |  |  |  |  |  |  |  |  |  |  |  |  |  |  |  |  |  |  |  |  |  |  |  |  |  |  |  |  |  |  |  |  |  |  |  | • | • |  |  |  |  |  |  |  |  |  |  |  | • |  |  |  |  |  |  |  |  |  |  |
| B8 |  |  |  |  |  |  |  |  |  |  |  | • |  |  |  |  |  | • |  |  |  |  |  |  |  |  | • | • |  |  | • |  |  |  |  |  |  | • |  |  |  |  |  |  |  |  |  |  |  |  |  |  |  |  |  |  |  |  |  |  |  | • • • |
| B9 |  |  |  |  |  |  |  |  |  | • |  | • |  |  |  |  |  |  |  |  |  |  |  |  |  |  |  |  |  |  |  |  |  |  |  |  | • |  |  | • |  |  |  |  |  |  |  |  |  |  |  |  |  |  |  |  |  |  |  |  |  |  |
| L1 |  |  |  |  |  |  |  |  |  |  | • |  |  |  |  |  |  |  |  |  |  |  |  |  |  |  |  |  |  |  |  |  |  |  |  |  |  |  |  |  |  |  |  |  |  |  |  |  |  |  |  |  |  |  |  |  |  |  |  |  |  |  |
| L2 |  |  |  |  |  |  |  |  |  |  |  |  |  |  |  |  |  |  |  |  |  |  |  |  |  |  |  |  |  |  |  |  |  |  |  |  |  |  | • |  |  |  |  |  |  |  |  |  |  |  |  |  |  |  |  |  |  |  |  |  |  |  |
| R1 | • |  |  |  |  |  |  |  |  |  |  |  |  |  |  |  |  |  |  |  |  |  |  |  |  |  |  |  |  |  |  |  |  |  |  |  |  |  |  |  |  |  |  |  |  |  |  |  |  |  |  |  |  |  |  |  |  |  |  |  |  |  |
| R2 |  |  |  |  |  |  |  |  |  |  |  |  |  |  |  |  |  |  |  |  |  |  | • |  |  |  |  |  |  |  |  |  |  |  |  |  |  |  |  |  |  |  |  |  |  |  |  |  |  |  |  |  |  |  |  |  |  |  |  |  |  |  |
| R3 |  |  |  |  |  |  |  |  |  |  |  |  |  |  |  |  | • |  |  |  |  |  |  |  |  |  |  |  | • |  |  |  |  |  |  |  |  |  |  |  |  |  |  |  |  |  |  |  |  |  |  |  |  |  |  |  |  |  |  |  |  |  |

**Appendix S1c.** Capture histories of all individuals detected in BPP.

| Study area | Parameter | Point estimate | Upper 95% confidence interval (CI) |
| --- | --- | --- | --- |
| SBNP | N | 1.01 | 1.03 |
|  | p0 | 1.00 | 1.01 |
|  | psi | 1.01 | 1.04 |
|  | Sigma | 1.01 | 1.01 |
| UCEZ | N | 1.01 | 1.02 |
|  | p0 | 1.00 | 1.00 |
|  | psi | 1.01 | 1.02 |
|  | Sigma | 1.01 | 1.03 |
| BPP | N | 1.00 | 1.01 |
|  | p0 | 1.00 | 1.00 |
|  | psi | 1.00 | 1.01 |
|  | Sigma | 1.01 | 1.01 |

**Appendix S2.** Gelman-Rubin diagnostic statistics for population size *N*, population augmentation parameter *psi*, detection probability *p0* and detection function scale *sigma* for all study areas


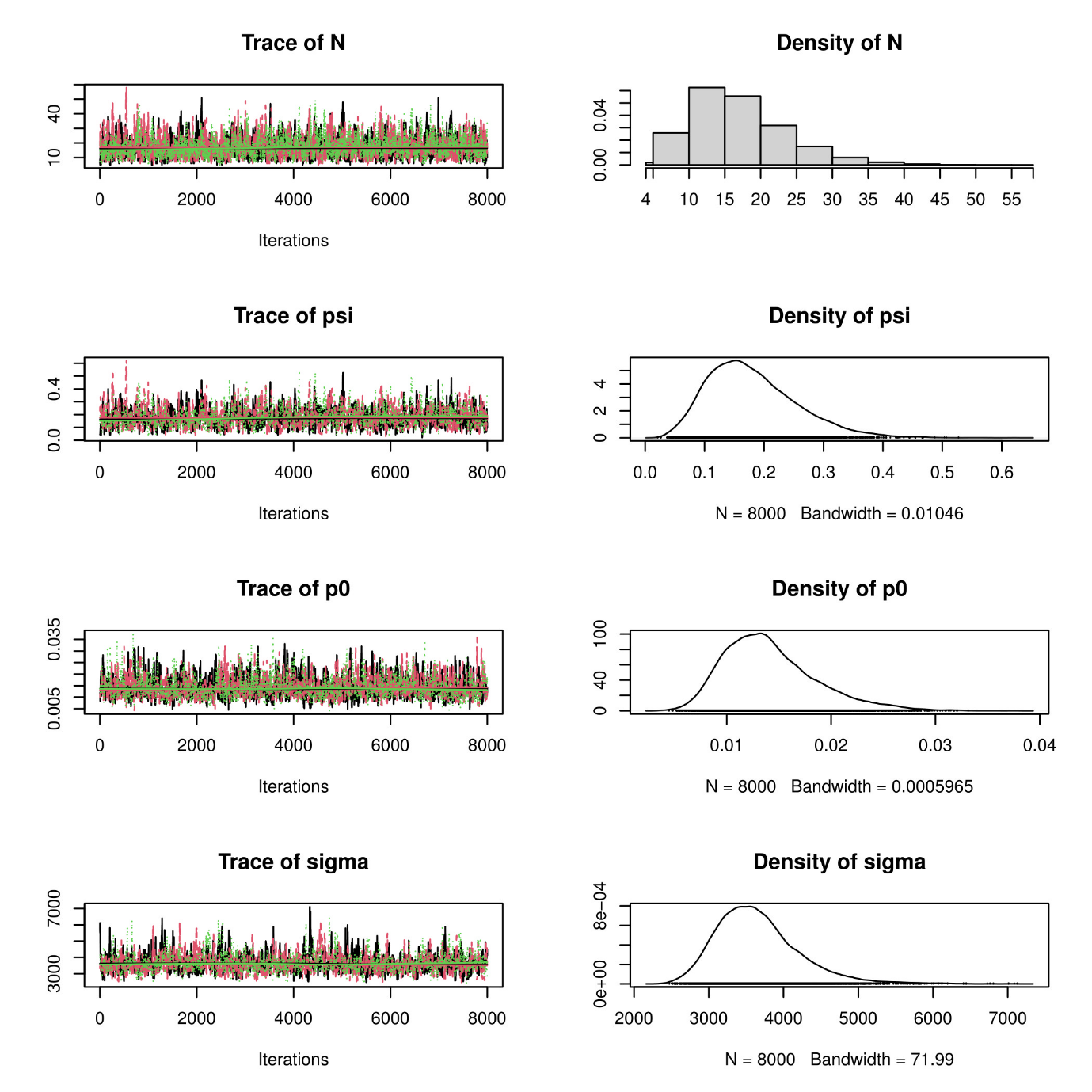
**Appendix S3a.** Iteration traces of the MCMC algorithm and posterior distributions of population size *N*, population augmentation parameter *psi*, detection probability *p0* and detection function scale *sigma* for the SBNP.

**
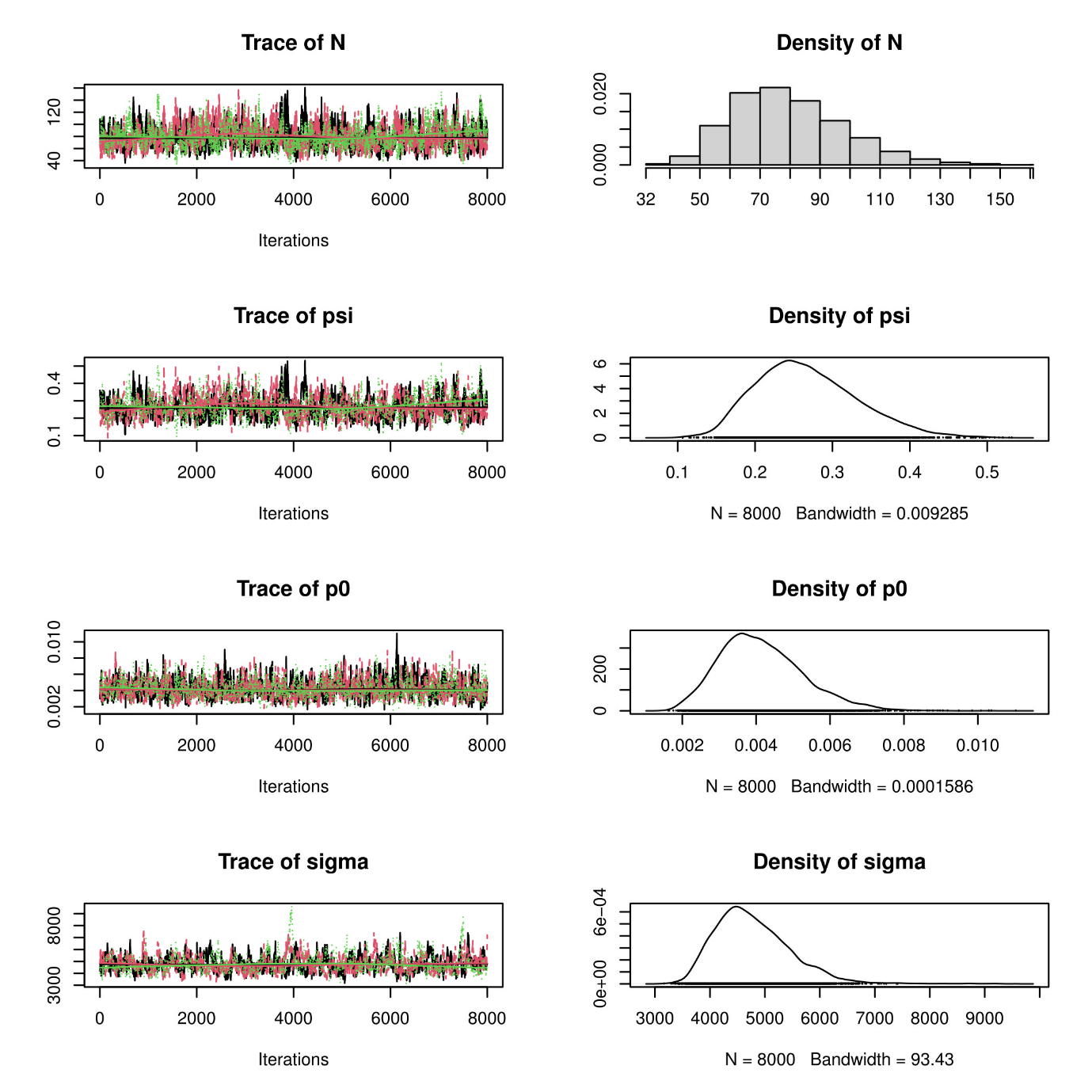
**

**Appendix S3a.** Iteration traces of the MCMC algorithm and posterior distributions of population size *N*, population augmentation parameter *psi*, detection probability *p0* and detection function scale *sigma* for the UCEZ.

**
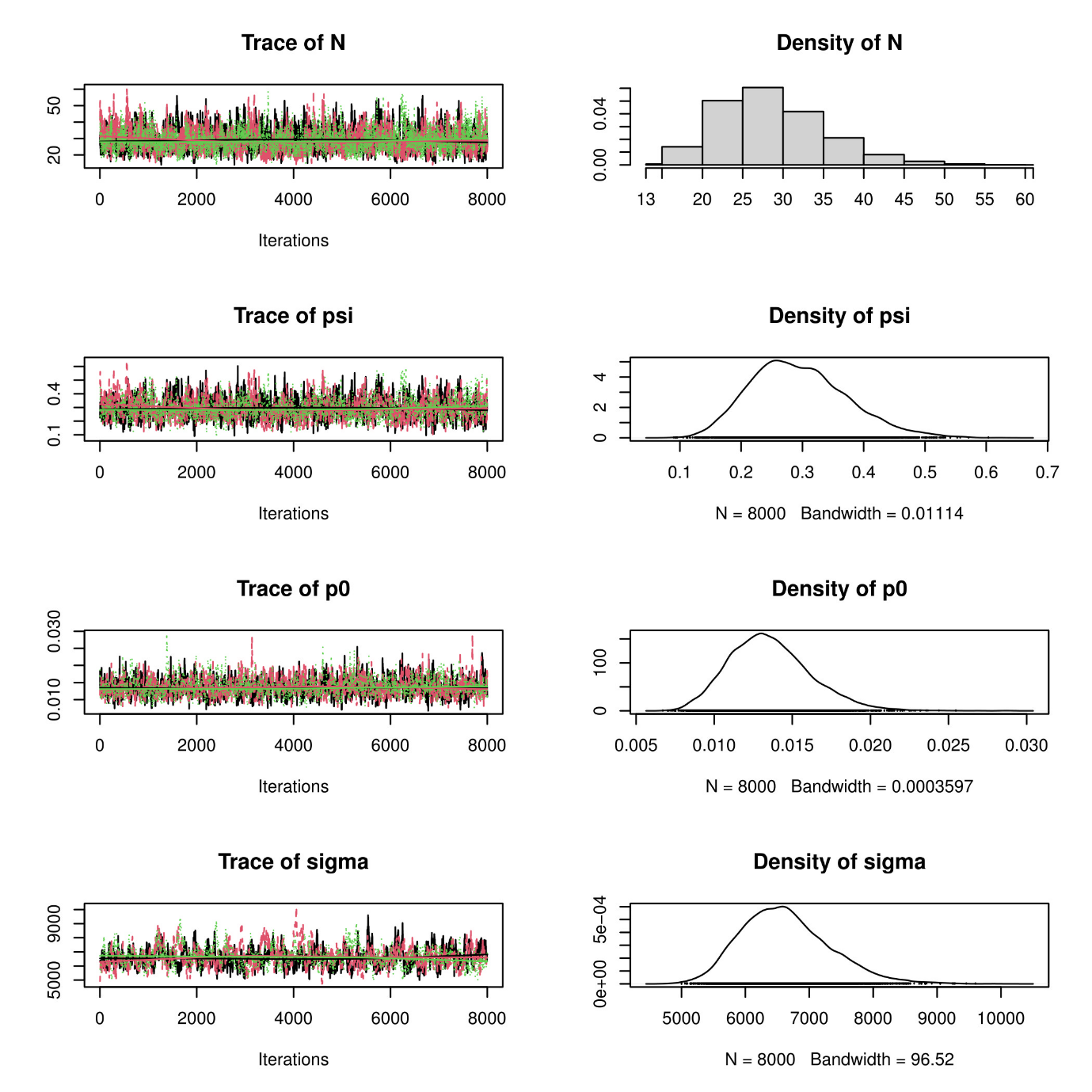
**

**Appendix S3a.** Iteration traces of the MCMC algorithm and posterior distributions of population size *N*, population augmentation parameter *psi*, detection probability *p0* and detection function scale *sigma* for BPP.
